# *Caenorhabditis elegans* Battling Starvation Stress: Low Levels of Ethanol Prolong Lifespan in L1 Larvae

**DOI:** 10.1371/journal.pone.0029984

**Published:** 2012-01-18

**Authors:** Paola V. Castro, Shilpi Khare, Brian D. Young, Steven G. Clarke

**Affiliations:** Department of Chemistry and Biochemistry and the Molecular Biology Institute, University of California Los Angeles, Los Angeles, California, United States of America; National Cancer Institute, United States of America

## Abstract

The nematode *Caenorhabditis elegans* arrests development at the first larval stage if food is not present upon hatching. Larvae in this stage provide an excellent model for studying stress responses during development. We found that supplementing starved larvae with ethanol markedly extends their lifespan within this L1 diapause. The effects of ethanol-induced lifespan extension can be observed when the ethanol is added to the medium at any time between 0 and 10 days after hatching. The lowest ethanol concentration that extended lifespan was 1 mM (0.005%); higher concentrations to 68 mM (0.4%) did not result in increased survival. In spite of their extended survival, larvae did not progress to the L2 stage. Supplementing starved cultures with n-propanol and n-butanol also extended lifespan, but methanol and isopropanol had no measurable effect. Mass spectrometry analysis of nematode fatty acids and amino acids revealed that L1 larvae can incorporate atoms from ethanol into both types of molecules. Based on these data, we suggest that ethanol supplementation may extend the lifespan of L1 larvae by either serving as a carbon and energy source and/or by inducing a stress response.

## Introduction

The nematode worm *Caenorhabditis elegans* is a powerful model organism for aging studies due to its short generation time and lifespan [1], [2]. Survival under conditions of stress can be enhanced by entering alternate larval stages, including diapauses in L1 larvae [3], [4], [5], dauer larvae [6], and L4 larvae [7]. In these alternate larval stages, worms are more stress resistant and have enhanced lifespans [3], [7], [8]. Dauer diapause, entered in response to the unfavorable stress conditions of starvation, increased temperature and overcrowding, has been most intensively studied [6], [9], [10], [11]. Dauer larvae feature a thicker cuticle and sealed buccal and anal cavities [6]. Dauer larvae are more resistant to oxidative [9], osmotic [10] and hypoxic stress [11].

In this work, we have focused on the L1 diapause that is induced by starvation upon egg hatching [3], [4], [5]. In this diapause, larvae are more stress resistant even though no morphological changes are observed [5], [12]. Entry into L1 diapause is regulated by insulin-like receptor DAF-2 [13], the canonical regulator of aging that also regulates developmental arrest in dauer diapause [14]. Both of these effects are mediated via the FOXO transcription factor DAF-16 [5], [15].

In previous work, *C. elegans* lacking the *pcm-1* encoded enzyme that can repair age-damaged proteins have been shown to have defective survival as both starved L1 larvae and dauer larvae [16], [17]. Supplementation of M9 medium with an ethanolic solution of cholesterol was shown to extend the lifespan of wild-type starved L1 larvae to a greater extent than *pcm-1* mutant L1 larvae [17]. However, it was not clear whether the longevity effects were due to cholesterol, ethanol, or a combination of the two compounds. In this study, we show that lifespan extension in starved L1 larvae is due to ethanol and not to cholesterol and have focused on the mechanism of ethanol action in extending longevity.

## Methods

### Worm strains and procedures

Standard procedures were used to maintain *C. elegans* strains [18]. Strains used included the wild-type N2 strain, the *pcm-1(qa201)* mutant strain backcrossed into the N2 background [16], and the pharyngeal pumping mutant *eat-2 (ad1116)*. The latter strain was obtained from the *Caenorhabditis* Genetics Center.

### L1 Survival Assay

Mixed stage worms were cultured at 20°C for 2 to 3 days on approximately 30 to 60 100 mm diameter agar plates. NGM agar plates [18] were used for the experiments comparing N2 and *pcm-1* larvae and for the experiment where ethanol was added at various times after incubation; HGM agar plates (NGM plates with eight times the amount of Bacto Peptone; Lab-Express, Ann Arbor, MI) were used for the other experiments. Plates were streaked with the streptomycin-resistant strain of *Escherichia coli* OP50. Nematodes (mostly gravid adults) were collected with M9 medium (22 mM potassium dihydrogen phosphate, 42.3 mM sodium monohydrogen phosphate, 85.6 mM NaCl, and 1 mM MgSO_4_) to yield about 2 ml of pelleted worms after centrifugation in a clinical centrifuge at room temperature. These worms were resuspended in 5 ml of 1.5% sodium hypochlorite/0.63 M NaOH and repelleted. The pellet was then resuspended in 10 ml of 1.5% sodium hypochlorite/0.63 M NaOH, vortexed for about 3 min to disrupt most of the adult worms, and repelleted. The resulting egg pellet was then suspended in 12 ml of M9 medium and washed four times to remove the residual bleaching solution. Eggs (10^6^ to 2×10^6^) were finally resuspended in 50 to 70 ml of M9 medium in 250 ml baffled glass culture flasks and incubated with shaking (160 rpm, 20°C) for 24 h to allow hatching into synchronized L1 larvae. Unless otherwise stated, about 70,000 L1 larvae were diluted to a final volume of 70 ml of M9 medium or 70 ml of M9 medium supplemented with various chemicals and further incubated under the same conditions. Supplemented materials include methanol (Fisher catalog no. A4524, HPLC, meets ACS specifications), ethanol (Fisher catalog no. AC615100010, 99.5%, anhydrous), n-propanol (Kodak catalog no. 1088699), isopropanol (Fisher catalog no. BP26324, 99.9%, HPLC Grade), n-butanol (Fisher catalog no. AC107690010, 99%), and isobutanol (Fisher catalog no. A3971, Certified ACS). Every 48 h, three 30 µl aliquots of the culture were pipetted on NGM plates and were scored for survival (motility or movement induced by platinum wire prodding); the percentage of live worms was determined following scoring of approximately 100 L1 larvae per condition. Statistical analysis of individual survival experiments was done using the Mantel-Cox logrank method.

### Assay of ethanol in culture media

An alcohol dehydrogenase assay [19] was used to measure the ethanol concentration in the culture medium over time. Alcohol dehydrogenase catalyzes the oxidation of ethanol to acetaldehyde with simultaneous reduction of NAD^+^ to NADH. The latter product can be detected by its absorbance at 340 nm. The rate of change of the absorbance at 340 nm is directly proportional to the ethanol concentration in the sample. Aliquots (48 µl) of culture supernatant (or mock culture lacking larvae) were removed every 2–3 days and incubated with 141 µl of 32 mM sodium pyrophosphate buffer pH 8.8, 93 µl of 6 mM **β-**NAD (Sigma, catalog no. N7004) and 18 µl of 0.2 mg/ml alcohol dehydrogenase from *Saccharomyces cerevisiae* (Sigma, catalog no. A3263) dissolved in 0.01 M sodium phosphate buffer at pH 7.5 with 0.01% gelatin. The reaction was carried out at room temperature in a 96-well plate using a plate reader model Spectra Max M5 (Molecular Devices). The initial rate of NAD^+^ reduction was obtained by allowing the reaction to proceed for 30 s, taking absorbance readings every 5 s. A standard curve was obtained by replacing the 48 µl of culture supernatant with 48 µl of M9 containing known ethanol concentrations ranging from 0.1 mM to 110 mM. To calculate the ethanol concentration in the culture, the absorbance rate of change obtained for the culture was interpolated into the standard curve.

### Fatty acid analysis by gas chromatography-mass spectrometry (GC-MS)

L1 larvae prepared as described above for the survival experiments were incubated for various times at 20°C at 160 rpm in M9 medium alone, M9 medium with 1 mM ethanol, or M9 medium with 1 mM fully-deuterated ethanol (Sigma, catalog no. 186414). Lipids were extracted and fatty acid methyl esters prepared after the procedure of Hill *et al.*, 2011 [20]. Larvae (concentrated by centrifugation to about 1 ml) were transferred to 5-ml borosilicate centrifuge tubes with screw caps and mixed with 1 ml of 20% (w/v) KOH in 1∶1 methanol:H_2_O and samples were boiled in a 100°C water bath for 1 h. Samples were then acidified to pH 2 with approximately 11 drops of 37.6% HCl. For fatty acid transesterification, 2 ml of 15% BF_3_ in methanol (Sigma) was added and samples were boiled for 5 min. Fatty acid methyl esters were then extracted by first adding 1 ml of a saturated NaCl solution and then 2 ml of 1∶4 CHCl_3_ (certified ACS, Fisher):hexanes (99.9%, Fisher). Samples were vortexed and the organic upper layer was collected. The extraction of the water phase was repeated with an additional 2 ml of 1∶4 CHCl_3_:hexanes. The combined organic layers containing fatty acid methyl esters were dried under a stream of nitrogen gas and resuspended in 200 µl of ethyl acetate. One µl was then taken for analysis on an Agilent 6890–6975 GC-MS instrument equipped with a DB-wax column (0.25-mm inner diameter×30-m length×0.25-µm film thickness (Agilent catalog no. 122-7032). The initial temperature for the column was set at 100°C. The temperature was increased to 200°C at a rate of 20°C/min and remained at this temperature for 10 min. The temperature was then increased to 250°C at a rate of 15°C/min and held constant for 5 min. The front inlet had a pressure of 16.69 psi. A split ratio of 5∶1 was used with a split flow of 5.0 ml/min and a total flow of 8.6 ml/min. MSD ChemStation Data Analysis Application software was used to obtain quantitative data from the mass spectra of the chromatogram peaks.

### Amino acid analysis by GC-MS

L1 larvae prepared as described above for the survival experiments (200,000 larvae/time point) were concentrated by centrifugation to 1 ml and frozen at −80°C before being thawed, washed twice with 1 ml of water and spun at 6800× g for 3 min. The larval pellets were resuspended in 1 ml of 40∶40∶20 acetonitrile∶methanol∶water and 100 µl of 0.5-mm glass beads (BioSpec Products) and kept on ice. A Mini-BeadBeater (BioSpec Products) was used to mechanically disrupt the worms with 15 cycles of a 30-s agitation followed by a 30-s rest. Cellular debris and precipitated protein was isolated by centrifugation at 20,800× g for 20 min at 4°C. Each pellet was resuspended in 125 µl of water and this mixture was transferred to 6×50-mm glass tubes. After addition of 125 µl of 25% trichloroacetic acid (Fisher), the tubes were incubated at room temperature for 30 min before centrifugation at 4,000× g for 30 min to precipitate protein. The protein pellets were washed with 100 µl of acetone at −20°C and spun again at 4,000× g for 30 min. To hydrolyze the precipitated protein, 100 µl of 12.1 N hydrochloric acid (Fisher) was added and the tubes were heated *in vacuo* at 110°C for 20 h (Waters Pico-Tag Vapor-Phase apparatus). After the incubation, each sample was dried by vacuum centrifugation, resuspended in 100 µl of water, and sonicated in a water bath for 20 min. After centrifugation at 4000× g for 5 min, the supernatants were transferred to glass vials and dried by vacuum centrifugation. The samples were then derivatized for GC-MS analysis by addition of 50 µl of 2% methoxyamine in pyridine (both from Sigma). After incubation at 60°C for 1 h, the samples were dried under N_2_ and 50 µl of *N,O*-bis(trimethylsilyl)trifluoroacetamide in 10% trimethylchlorosilane (Pierce) was added. Following another 1-h incubation at 60°C, each sample was analyzed twice by GC-MS. Aliquots (1-µl) of the derivatized samples were injected (splitless mode, Agilent Technologies 6890 gas chromatograph) onto a medium polarity bonded phase fused silica capillary column (60-m×0.25-mm internal diameter, 0.2-µm film thickness, 5% phenyl-95% ethyl, HP-5 ms, Agilent Technologies) using helium as the carrier gas (1 ml/min, constant flow). The column was attached to the electron impact ion source (70 eV, 180°C) of a constantly scanning (m/z = 50–600, 1 scan/sec) time-of-flight mass spectrometer (Micromass GCT, Waters). The GC injector port and the GC-MS transfer line were maintained at 250°C, and the GC oven was held at 50°C for 3 min following injection and increased linearly at 5°C/min to a plateau of 300°C. The data were analyzed using the MassLynx software suite (Waters). The approximate retention profile of each amino acid was determined by searching the spectra of each peak on the total ion chromatogram against the NIST Mass Spectral Library. These assignments were verified by comparing the spectra and retention profiles of the samples and those of a similarly derivatized amino acid standard mix (1270 µM for each amino acid) (Standard H, Pierce). A QuanLynx processing method was developed to quantify unlabeled and labeled forms of each amino acid using the M-15 ion, except lysine where the M peak was used. For several amino acids that appeared to incorporate the labeled ethanol, the M-117 ion was also analyzed. Labeled forms were only included in our analysis where the ions were abundant enough to allow for straightforward integration. For each amino acid in each sample, relative fractions of the unlabeled and various labeled species were calculated. These fractions were averaged and the standard deviation of the fractions for experimental and technical replicates was calculated. Significance was assessed with a Student's T-test of the calculated fractions in the 0-day samples and the 3 and 7-day samples.

## Results

**Figure 1:**
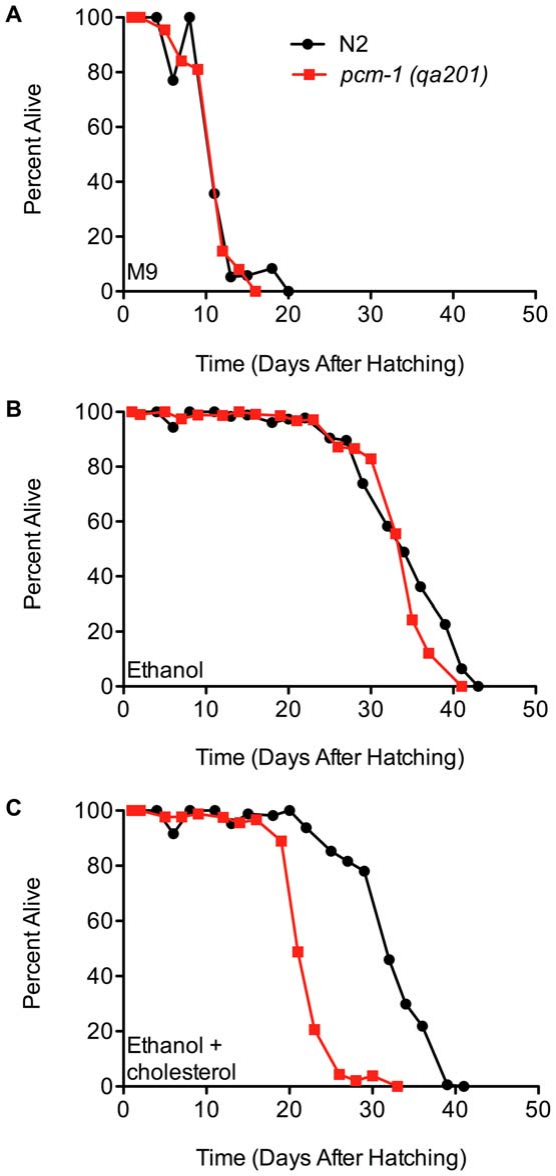
Low concentrations of ethanol extend lifespan of arrested wild-type (N2) and *pcm-1* mutant L1 larvae. Following hypochlorite treatment of wild-type N2 (black line) or *pcm-1* mutant (red line) gravid adults, eggs were isolated and incubated in M9 medium for 24 h at 20°C on a rotary shaker at 160 rpm as described in the “Methods” section. The resulting starved L1 larvae (300 N2 larvae/ml or 1000 *pcm-1* larvae/ml) were then incubated at 20°C at 160 rpm in either 70 ml of M9 medium (panel A) or 70 ml of M9 medium supplemented with 17 mM ethanol (0.1%; panel B) or 17 mM ethanol and 13 µM cholesterol (5 µg/ml; panel C) and survival was scored every 48 hours until all larvae were dead. Using the Mantel-Cox logrank test, a p-value of 0.03 was found for the significance of the difference between the *pcm-1* and N2 strains in panel B and a p-value of <0.0001 for these strains in panel C.

Addition of 13 µM cholesterol to starving L1 larvae incubated in M9 medium was previously reported to significantly increase their survival [17]. However, since the cholesterol was dissolved in ethanol, resulting in a final solvent concentration of 17 mM (0.1%), we wanted to ask if ethanol itself might be responsible for some or all of the lifespan extension. We thus repeated the previous experiments with an additional control of ethanol alone. We found that starved wild-type L1 larvae incubated in M9 medium live about 15 days (Fig. 1, panel A), while larvae incubated in M9 medium supplemented with cholesterol dissolved in ethanol live about 30 days, or about twice as long (panel C). These results are similar to those previously reported [17]. However, the lifespan of N2 L1 larvae was also extended to about 30 days when only ethanol was added to the M9 medium (panel B), suggesting that the observed effect of cholesterol can be attributed to the ethanol solvent. We also repeated these experiments in the protein repair deficient *pcm-1* strain. Here, we observed similar starvation survival in the presence and absence of 17 mM ethanol as the wild-type N2 strain (Fig. 1, panels A and B). But significantly, the survival with 13 µM cholesterol/17 mM ethanol was greatly reduced in the *pcm-1* mutant compared to 17 mM ethanol alone (panel C). These experiments are summarized in Table S1. These results suggest that cholesterol may be toxic to *pcm-*1 mutants, rather than leading to a partial extension of lifespan as originally reported [17].

**Figure 2:**
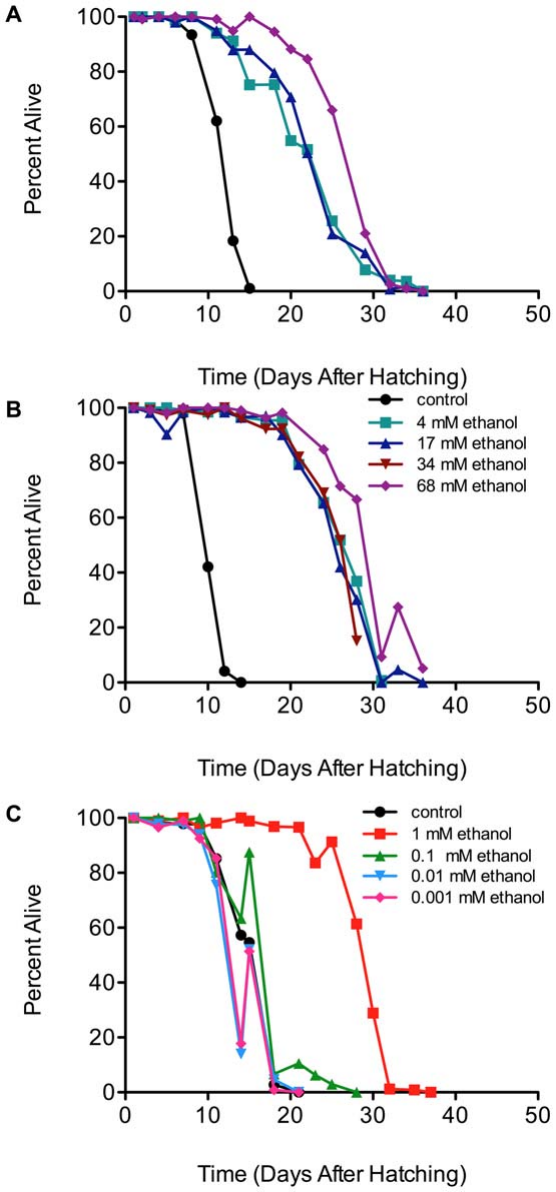
Small concentrations of ethanol (1 mM–68 mM) extend the lifespan of arrested wild-type L1 larvae. L1 larvae (70,000) were isolated as described in Figure 1 and were incubated at 20°C in either M9 medium only or M9 medium supplemented with 4 mM, 17 mM, or 68 mM ethanol (panels A and B; legend for both in panel B) and 0.001, 0.01, 0.1, or 1 mM ethanol (panel C). Survival was scored every 48 hour until all larvae were dead. Mantel-Cox logrank analyses gave a p-value of <0.0001 for the difference between the control (no ethanol) and all concentrations of ethanol at 1 mM or above. The survival at 68 mM ethanol is significantly different from that at 17 mM ethanol in panel A (p = 0.0001) and in panel B (p = 0.001).

To characterize the lifespan extension due to ethanol, we first investigated the concentration dependence of the effect. We found that ethanol concentrations ranging from 4 mM to 68 mM (0.024% to 0.4%) result in similar lifespan extensions to the one obtained with 17 mM (0.1%) ethanol (compare Fig. 1, panels A and B, with Fig. 2, panels A and B). Interestingly, we noted no large differences in survival at the higher (or lower) ethanol concentrations. We have summarized these data in terms of 75%, 50%, and 25% survival in Table S1 and show the statistics for replicate experiments in Table S2. To determine the lowest ethanol concentration that would extend lifespan, we incubated L1 larvae in ethanol concentrations ranging from 0.001 to 1 mM (Fig. 2, panel C). Ethanol concentrations below 1 mM (0.1, 0.01 and 0.001 mM) did not result in the clear lifespan extension (Fig. 2, panel C) that we observed with concentrations ranging from 4 to 68 mM (Fig. 2, panels A and B). These results suggest that very low (1 mM) ethanol concentrations are effective in prolonging the starvation survival of L1 larvae.

In spite of the enhanced survival of L1 larvae in M9 medium supplemented with ethanol, we found no evidence that these larvae could escape from the developmental arrest diapause. There is a considerable increase in body length when L1 larvae become L2 larvae (a change from about 250 µm to 350 µm [6], [21], [22]). We found no increase in body length over time for L1 larvae starved with or without 4 mM ethanol, indicating that the L1 larvae do not progress to L2 larvae (or more developmentally advanced larvae/adults) under these conditions (Figs. S1 and S2). L1 larvae incubated with 4 mM ethanol, however, appear robust and healthy up to day 21, while L1 larvae incubated in M9 medium alone are generally dead by day 14 with commonly seen detached cuticles (Fig. S2).

**Figure 3:**
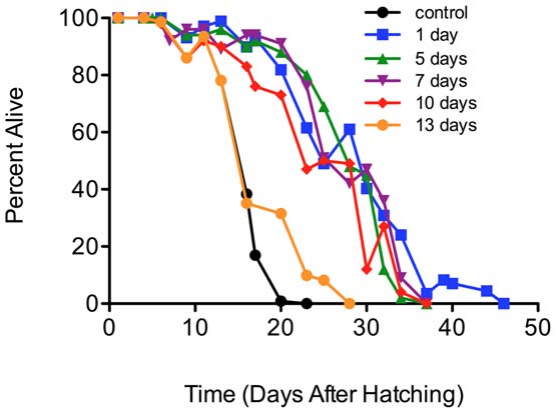
Ethanol added to M9 medium up to 10 days post-hatching extends lifespan of arrested wild-type L1 larvae. Wild-type L1 larvae (70,000) were isolated as described in Figure 1 and were transferred to 70 ml of M9 medium supplemented with ethanol (4 mM) after 1, 5, 7, 10 and 13 days of incubation under starvation conditions in M9 medium at 20°C and 160 rpm. Survival was scored every 48 hours until all larvae were dead. Mantel-Cox logrank analyses indicated that the control was significantly different from the 1, 5, 7, and 10 day samples (p<0.0001) and the 13 day sample (p = 0.01).

In order to identify when during the L1 larvae incubation the presence of ethanol was important for starvation survival, L1 larvae were incubated in M9 media that was supplemented with ethanol at various time points (one, five, seven, ten and thirteen days) after the cultures were started. We found that the ethanol added up to 10 days after starvation resulted in lifespan extension of the L1 larvae (Fig. 3, Table S1). These results suggest that the beneficial effect of the ethanol can occur up to 10 days of starvation and to a lesser extent up to 13 days of starvation.

**Figure 4:**
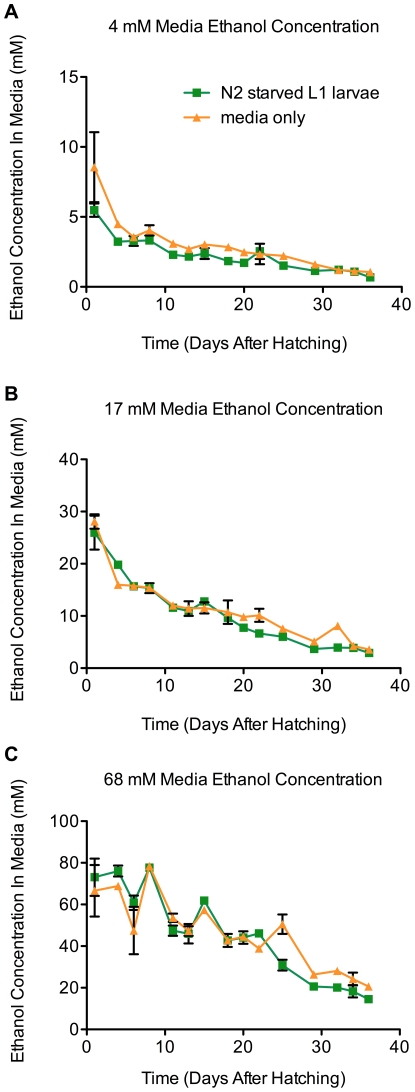
Concentrations of ethanol in incubated M9 medium and in M9 medium with arrested wild-type L1 larvae. L1 larvae (70,000) were isolated as described in Figure 1 and were incubated at 20°C at 160 rpm in 70 ml of M9 medium supplemented with 4 mM, 17 mM, and 68 mM ethanol (panels A, B, and C, respectively). The concentration of ethanol in the medium with L1 larvae (green curve) and a control medium without larvae (orange) was measured over time as described in the “Methods” section. Measurements were taken until all larvae were dead. The error bars correspond to the standard deviation of triplicate assays. We note the small differences between the nominal concentrations of ethanol added and the initial concentrations detected - the origin of this difference is not clear.

We expected that ethanol levels in the culture medium might decrease from both evaporation into the environment and by possible consumption by the L1 larvae. To ask how much ethanol remains in the medium over the course of incubation, and whether the L1 larvae might be capable of taking up ethanol from the culture, we measured the concentration of ethanol in the medium at various incubation times with and without wild-type L1 larvae. We did this for three replicate cultures with starting concentrations of 4 mM, 17 mM and 68 mM of ethanol (Fig. 4). We found that the concentration of ethanol in the culture with worms and the control mock culture decreased at similar rates. These results indicate that ethanol persists in the cultures and is not taken up by the larvae in large quantities. For example, there was approximately 1, 3 and 13 mM ethanol left in the cultures supplemented with 4, 17 and 68 mM ethanol, respectively, after 35 days of incubation when all larvae were dead (Fig. 4). We did, however, obtain evidence that there may have been some uptake of the ethanol by the worms in the 4 mM ethanol culture where the ratio of larvae to ethanol was the highest (Fig. 4, panel A). Here, we noted less ethanol at each time point in the incubation in the cultures where larvae were present compared to the mock culture medium. It should be noted that low levels of ethanol uptake into the larvae may not be detectable as a loss of ethanol in the media.

**Figure 5:**
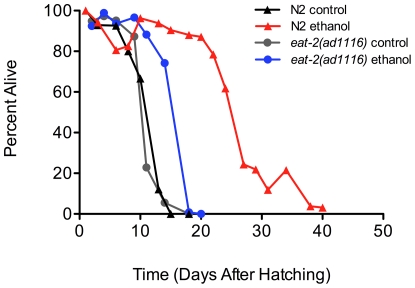
Intact pharyngeal pumping machinery is necessary for full ethanol-mediated lifespan extension of arrested wild-type L1 larvae. Wild-type (N2) and *eat-2(ad1116)* mutant L1 larvae (70,000) were prepared as described in Figure 1, and were transferred to 70 ml of M9 medium only (wild-type (black line), *eat-2* (gray line)) or 70 ml of M9 medium supplemented with 4 mM ethanol (wild-type (red line), *eat-2* (blue line)) and survival at 20°C was scored every 48 hours until all larvae were dead. Mantel-Cox logrank analyses showed that lifespan was significantly different in the wild-type and *eat-2* mutants in the presence of 4 mM ethanol (p<0.0001).

These results prompted us to ask if L1 larvae were capable of internalizing ethanol by pharyngeal pumping. To test this hypothesis we incubated pharyngeal pumping defective *eat-2 (ad1116)* L1 larvae in 4 mM ethanol and measured their starvation survival. These mutants are defective in a pharyngeal nicotinic acetylcholine receptor subunit required for a wild-type pumping rate. These mutant worms have a significantly reduced pumping rate [23] and are used in dietary restriction studies [24], [25]. We found that *eat-2 (ad1116)* mutant L1 larvae incubated in M9 had similar survival to wild-type (Fig. 5, Table S1). However, *eat-2 (ad1116)* mutant L1 larvae did not survive as long as wild-type N2 larvae when supplemented with 4 mM ethanol (Fig. 5, Table S1). This suggests that it is necessary for the worms to pump in ethanol for full lifespan extension during starvation, although it is possible that the defective acetylcholine receptor blocked the ethanol effect by a different mechanism.

**Figure 6:**
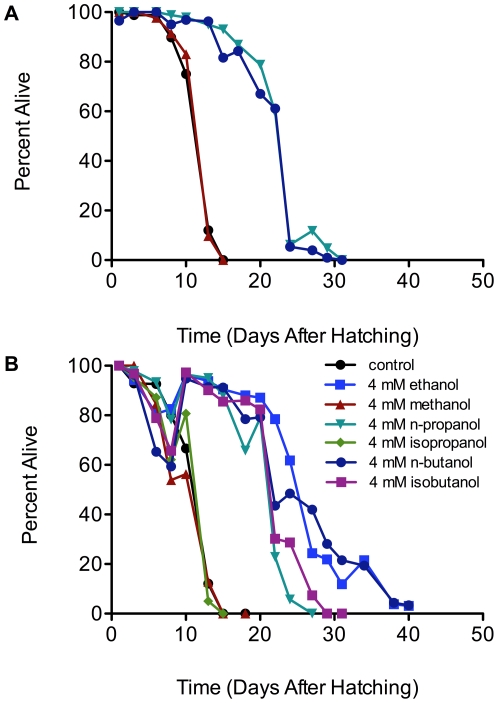
Alcohols containing 3 and 4 carbons can extend the lifespan of arrested wild-type L1 larvae. Survival was analyzed for wild-type L1 larvae (70,000) incubated at 20°C and 160 rpm as described in Figure 1 in either 70 ml of M9 medium only, or 70 ml of M9 medium supplemented with 4 mM methanol, n-propanol, or n-butanol (panel A), or M9 medium only, or M9 medium supplemented with 4 mM ethanol, methanol, n-propanol, n-butanol, isopropanol, or isobutanol (panel B). Survival was scored every 48 hours until all nematodes were dead. Mantel-Cox logrank analyses demonstrated significance (p<0.0001) for the lifespan difference of all alcohols versus controls except for methanol (p = 0.58 in panel A and p = 0.28 in panel B) and isopropanol (p = 0.43).

To analyze the biochemical specificity of the ethanol effect, we asked whether incubating L1 larvae in other alcohols would also result in lifespan extension. In a first experimental trial, we found that incubation with 4 mM concentrations of longer-chain alcohols, n-propanol and n-butanol, resulted in lifespan extension while the same concentration of methanol did not (Fig. 6, Table S1). In a second trial, we tested isopropanol and isobutanol in addition to methanol, n-propanol and n-butanol, all at 4 mM concentrations. We show the statistical analysis for replicate experiments in Table S2. Consistent with the first trial, we found that n-propanol and n-butanol resulted in lifespan extension similar to that of ethanol, while methanol did not result in lifespan extension. Isobutanol, but not isopropanol, was found to extend starvation survival. Since pathways for the conversion of ethanol and n-propanol to central metabolic intermediates via oxidation to acetyl-CoA and propionyl-CoA are known [26], [27], these results suggest that L1 larvae may be able to use these alcohols as carbon and energy sources. The metabolism of the other alcohols by *C. elegans* has not been studied to our knowledge, but the oxidation of n-butanol to n-butryl-CoA seems reasonable.

**Figure 7:**
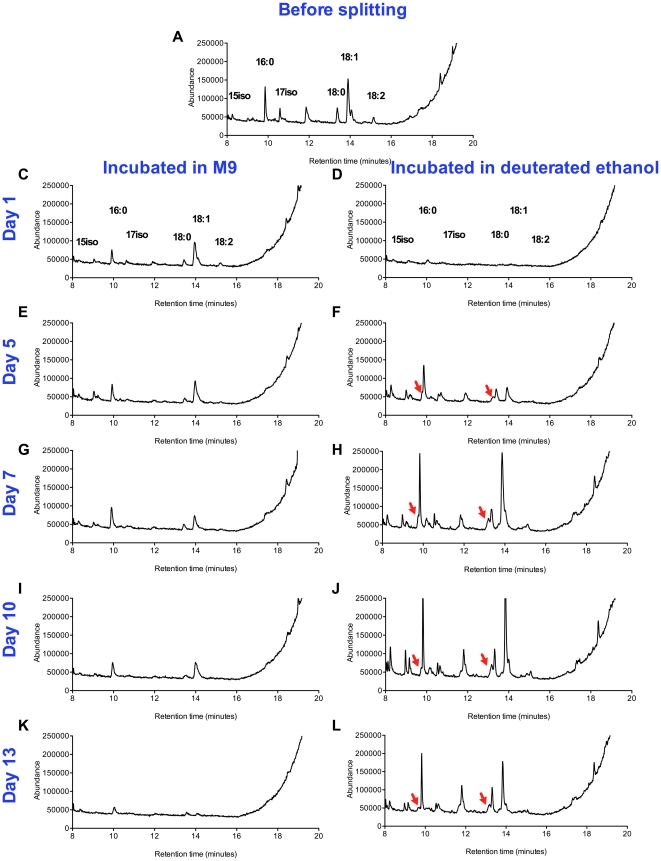
Fatty acid profile of arrested wild-type L1 larvae incubated in M9 or deuterated ethanol over time. Starved L1 larvae (100,000) were incubated in 100 ml of M9 only or 100 ml of M9 supplemented with 1 mM deuterated ethanol (C_2_D_5_OD) medium, under conditions described in Figure 1, and collected at six different time points. For each time point L1 larvae (100,000) were collected and total fatty acids analyzed after saponification and conversion to fatty acid methyl esters as described in the “Methods” section. Fatty acid methyl esters were fractionated and analyzed by GC-MS to look for the incorporation of deuterium atoms from the deuterated ethanol. Panel A shows the chromatogram for L1 larvae one day after hatching and before fully-deuterated ethanol was added. Panels C, E, G, I and K show the lipid profile for L1 larvae incubated in M9 medium for 1, 5, 7, 10 and 13 days, respectively. Panels D, F, H, J and L show the lipid profile for L1 larvae incubated in deuterated ethanol for 1, 5, 7, 10 and 13 days, respectively. Red arrows indicate the peaks containing deuterated fatty acids.

**Figure 8:**
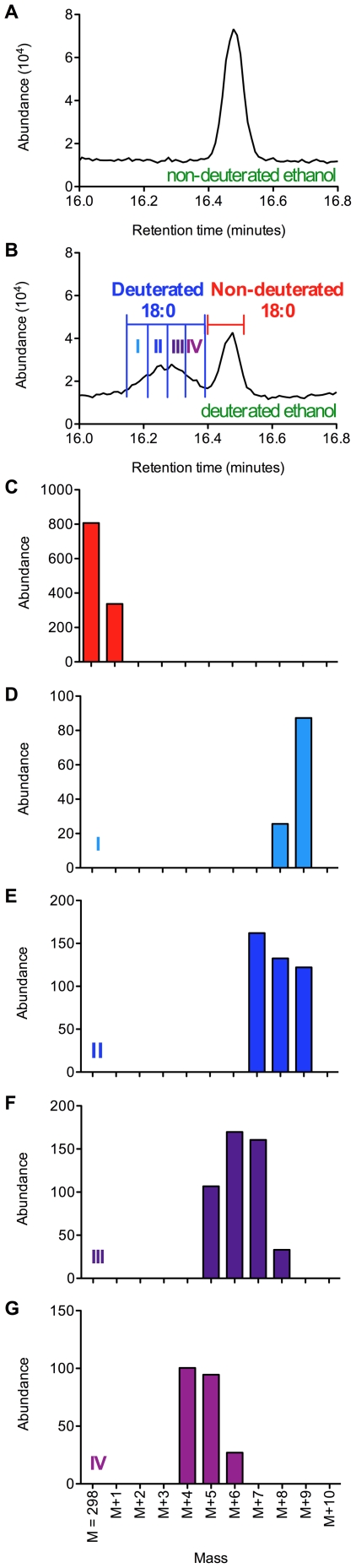
Arrested wild-type L1 larvae incorporate deuterium from deuterated ethanol into fatty acids. (panel A) A portion of the GC-MS chromatogram showing the peak of stearic acid methyl ester obtained form starved L1 larvae incubated for 7 days in 1 mM non-deuterated ethanol. (panel B) A similar portion of the GC-MS chromatogram showing deuterated (left) and non-deuterated (right) peaks of stearic acid for L1 larvae incubated for 7 days in deuterated ethanol. Deuterated fatty acids have shorter retention times and elute in a wide peak immediately before non-deuterated fatty acids. Panels D–G show the peak of deuterated stearic acid broken down into four quartiles as shown in panel B. Stearic acid methyl esters containing more deuterium atoms elute before stearic acid methyl esters containing fewer deuterium atoms. (panel D) Stearic acid in quartile I contains 9–10 additional mass units corresponding to 9–10 deuterium atoms. (panel E) Stearic acid in quartile II contains 7–9 additional mass units corresponding to 7–9 deuterium atoms. (panel F) Stearic acid in quartile III contains 5–8 additional mass units corresponding to 5–8 deuterium atoms. (panel G) Stearic acid in quartile IV contains 4–6 additional mass units corresponding to 4–6 deuterium atoms. The non-deuterated peak shows additional mass due to natural abundance of ^13^C (panel C).

**Figure 9:**
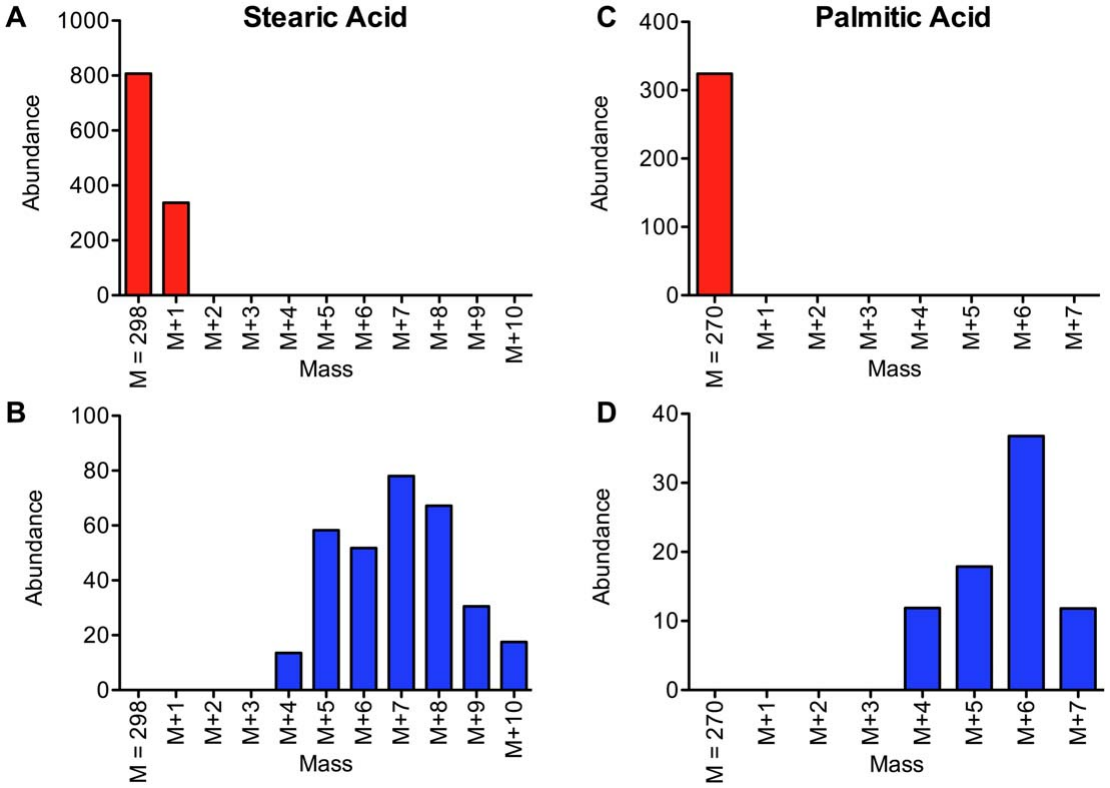
Arrested wild-type L1 larvae incorporate medium ethanol into stearic and palmitic acids. The isotopic distribution of stearic acid methyl ester and palmitic acid methyl ester from L1 larvae incubated for 7 days in 1 mM fully-deuterated ethanol is shown for the non-deuterated “sharp” peak (panels A and C) and for the earlier-eluting broad deuterated peak (panels B and D) (compare with Figure 8, panel B). Isotopic analysis of the broad peaks shows deuterium incorporation into stearic acid methyl ester that results in addition of 4–10 mass units (panel B) not seen in non-deuterated peak (panel A). Similarly, the earlier-eluting peak for palmitic acid methyl ester shows deuterium incorporation that results in addition of 4–7 mass units (panel D) not seen in the non-deuterated material (panel C).

To test the hypothesis that L1 larvae extend their survival by converting ethanol to acetate and fatty acids, perhaps as a mechanism to store fuel, we incubated the worms in fully deuterated ethanol and looked for deuterium incorporation into fatty acids using gas chromatography/mass spectrometry (GC-MS). As a control we measured the lifespan of starved L1 larvae incubated in 1 mM deuterated ethanol and found it to be similar to L1 larvae incubated in 1 mM ethanol (Fig. S3). In a first trial, we looked for incorporation of deuterium from ethanol into fatty acids after 1 and 7 days of incubation in deuterated ethanol (Figs. S4, S5, and S6). In a second trial, we looked for deuterium incorporation from ethanol into fatty acids at 5 different time points: after 1, 5, 7, 10 and 13 days of incubation in 1 mM deuterated ethanol (Fig. 7). In both trials, the chromatograms obtained from worms that were incubated in deuterated ethanol showed additional peaks eluting just before the peaks corresponding to several of the non-deuterated fatty acid methyl ester species (Fig. 7, Fig. 8 and Figs. S4, S5, and S6). These additional peaks elute in the positions expected for the deuterated derivatives [20], suggesting that these early peaks may correspond to the incorporation of deuterium atoms from ethanol into fatty acids. We confirmed this possibility by analyzing the mass spectra of the material in the early peaks. Mass spectrometry shows that the sharp peak eluting approximately at 16.5 min in the chromatogram from the extract of L1 larvae incubated with ethanol (Fig. 8, panel A) corresponds to stearic acid methyl ester. In Figure 8, panel B, we show a similar portion of the chromatogram from larvae incubated with fully-deuterated ethanol. The latter chromatogram has an additional broad peak eluting between 16.1 and 16.4 min. In Figure 8, panel C, we show molecular ions from the later-eluting peak of panel B correspond to non-deuterated stearic acid methyl ester. However, the broad peak in panel B contains a variety of deuterated derivatives of stearic acid methyl ester where additional masses corresponding to 4 to 9 daltons are present, indicating the incorporation of multiple molecules of deuterated ethanol (Fig. 8D–G). Figure 9 shows the molecular ion masses of the stearic acid from the non-deuterated peak and broad deuterated peak (Fig. 9, panels A and B), and also shows similar results for palmitic acid (Fig. 9, panels C and D). The early peak for stearic acid has an additional 4–10 mass units not seen in the non-deuterated peak. Similarly, the early peak for palmitic acid has 4–7 additional mass units not seen in the non-deuterated peak. In summary, we show that starvation-stressed L1 larvae incorporate deuterium atoms from ethanol into fatty acids, indicating that these larvae are capable of taking up ethanol from the medium and converting it to useful biomolecules.

**Figure 10:**
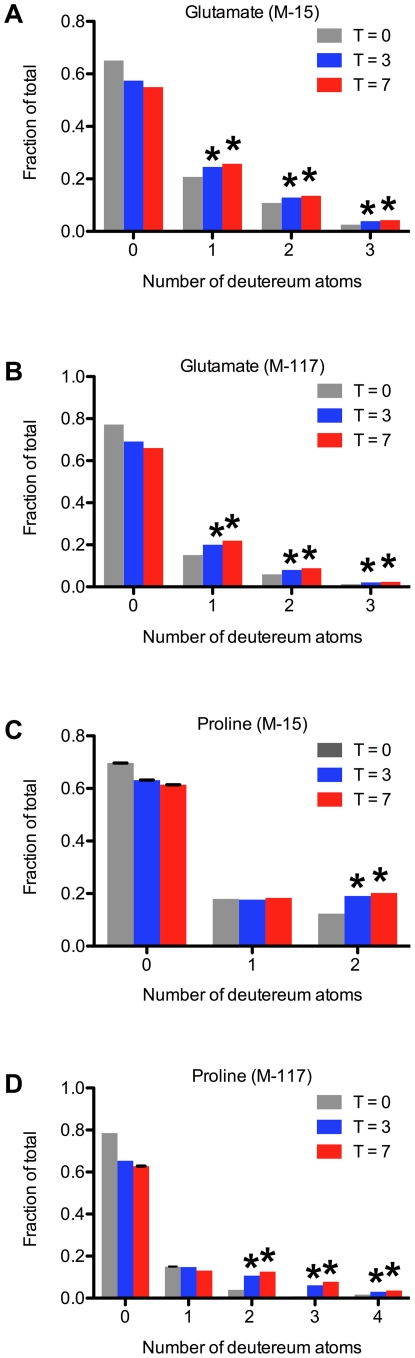
Arrested wild-type L1 larvae incorporate medium ethanol into protein-bound glutamate and proline. L1 larvae were isolated as described in Figure 1. For each time point (T = 0, 3 and 7 days) two 100-ml cultures containing 1000 starved L1 larvae/ml were prepared in triplicate. The zero day time point consisted of starved L1 larvae one day after hatching; the 3 day and 7 day time points consisted of larvae incubated with 1 mM fully-deuterated ethanol. Protein was isolated and hydrolyzed and amino acids were derivatized and analyzed using GC-MS for deuterium incorporation as described in the “Methods” section. The M-15 and M-117 ions for glutamate and proline were analyzed and data are shown as the average of 3 separate trials analyzed twice by GC-MS. Asterisks indicate a p-value from Student's T-test of less than 0.05 when the 3-day and 7-day peaks were compared to the 0-day controls.

To determine whether ethanol might also be incorporated into proteins via intermediates of the citric acid cycle, we examined the isotopic distribution of protein-derived amino acids from L1 larvae incubated in deuterated ethanol. Proteins were prepared from untreated larvae (t = 0 days), or larvae that were incubated with 1 mM fully-deuterated ethanol for 3 days or 7 days. GC-MS analysis of hydrolyzed protein showed a significant incorporation of deuterium into glutamate and proline (Fig. 10). We did not find significant deuterium incorporation in the other amino acids as shown in Figure S7. The glyoxylate cycle is induced in L1 larvae [28], providing a pathway that can convert acetyl-CoA from ethanol into intermediates of the citric acid cycle. Both glutamate and proline are direct products of alpha-ketoglutarate in the citric acid cycle. The incorporation of deuterium into the other amino acids may have not been detected due to dilution by unlabelled metabolic intermediates. Thus, ethanol can be converted into not only fatty acids, but also amino acids and potentially other metabolites.

## Discussion

We have shown here that very low concentrations of ethanol (1 mM, or 0.005%) can double the lifespan of starved L1 larvae of *C. elegans*. We demonstrate that these larvae can take up and incorporate ethanol into their fatty acids and amino acids. Although it does extend lifespan, ethanol does not replace food because we observed no developmental progression to the next larval stage in ethanol-treated L1 larvae. At this point, we do not know if the lifespan extension is due to a sensory response of the L1 larvae that inures them to starvation stress, or whether it is due to the use of ethanol as a carbon and energy source, or to a combination of both effects. Interestingly, we find that lifespan extension appears to be nearly maximal at 1 mM ethanol and does not markedly increase at concentrations up to 68 mM. These results suggest the saturation of a receptor system at about 1 mM ethanol or the possibility that a very low concentration of ethanol is needed for metabolism. Increased *de novo* fatty acid synthesis was previously observed in several long-lived adult *daf-2* mutants, suggesting a possible connection of increased fat stores with survival [29]. Our result that ethanol is still effective in lifespan extension when added up to 10 days after hatching suggests that L1 larvae are well-protected for a significant time under starvation, but that ethanol can increase this period of protection even when added late in the process. Since starvation conditions may be typical of many worm environments, the presence in water and soil of small amounts of ethanol formed from the fermentation of microorganisms [30], [31] may be important to the survival of the species in nature.

Previous studies of the effects of ethanol on *C. elegans* have generally used adult worms with much higher concentrations of ethanol than those we tested here with L1 larvae (1–68 mM), and have often focused on toxic effects. For example, adult feeding behavior is inhibited at ethanol concentrations greater than 340 mM [32], while development is slowed above 400 mM [33]. Exposing adult worms to increasing concentrations of ethanol (50–300 mM) results in a concentration-dependent depression of pharyngeal pumping rate [34]. However, a recent study found that 170–340 mM ethanol resulted in a small (less than 15%) increase in adult lifespan, while 680–850 mM ethanol resulted in a 30% or larger decrease in adult lifespan [35].

Other previous studies examining the effects of ethanol in *C. elegans* have focused on the detrimental effects on the nervous system of adults, with the aim to understand ethanol addiction and withdrawal in humans. The simplicity of the nervous system of *C. elegans* [36] has made it an attractive model organism for studying the depressive effect of high ethanol concentrations. A complete understanding of an organism's response to ethanol has not been fully elucidated since ethanol is a promiscuous molecule that interacts with many molecules in the nervous system, both pre- and post-synaptically [37]. Several mutants in *C. elegans* have provided information on the targets of ethanol in this organism. *C. elegans* lacking the BK potassium channel (*slo-1*) are resistant to the effects of 100–500 mM ethanol decreasing locomotion speed and body-bend amplitude [38]. Another study showed that adult worms lacking a membrane fusion regulator, *unc-18*, are resistant to the depressive effects of ethanol on locomotion when exposed to 300–500 mM ethanol [39]. Interestingly, worms treated at a much lower concentration of ethanol (20 mM) demonstrated enhanced locomotion. Finally, adult worms lacking a vesicle-associated GTPase (RAB-3) are resistant to the depressive effects of 200–400 mM ethanol [40]. It is not clear whether these targets of ethanol are involved in the lifespan extension observed here after supplementing cultures of L1 larvae with ethanol.

In the present study, we find that starved L1 larvae incorporate ethanol into fatty acids. Fatty acid synthesis has been previously found to be essential during L1 diapause [41]. Double mutants in Δ9 desaturase, an enzyme which produces monounsaturated fatty acids from saturated fatty acids, were shown to have reduced survival at low temperature and during L1 starvation [41]. The same study summarized the fatty acid composition of starved L1 larvae at hatching and 4 days after hatching [41]. Our data now extend these results to the period from 1 to 13 days after hatching, and provides additional support for a role of fatty acid synthesis in L1 survival during starvation.

Finally, our results emphasize the point that solvent molecules can have significant effects on biological systems. Interestingly, another solvent molecule, DMSO, used in many aging studies as a control, was recently shown to result in *C. elegans* adult lifespan extension in a *sir-2.1* and *daf-16* dependent manner [42].

## Supporting Information

Figure S1 **Starved L1 larvae incubated in the presence of ethanol do not progress to the L2 stage.** To confirm the staging of L1 larvae, body length was measured by differential interference contrast microscopy using the ZEISS Axio Imager.M1 with Hamamatsu ORCA-ER digital camera C4742-80. For each condition and time point approximately 20 L1 larvae were measured using Volocity 5 5.3.2 software. In three separate replicates, L1 larvae starved in M9 medium were examined one day after hatching and then after incubation in M9 medium or M9 medium supplemented with 4 mM ethanol for 9–10 days, 14–15 days, 21 days, and 28–29 days. For day 21 and 28 or 29 only larvae incubated in ethanol were analyzed since larvae incubated in M9 were no longer alive. Previous work has shown that L1 larvae measure close to 250 µm [6], [21], [22].
(TIFF)

Figure S2 **Wild-type L1 larvae incubated in 4 mM ethanol in M9 medium conserve healthy morphology longer than larvae starved in M9 medium alone.** Micrographs are shown of the larvae from the replicate 1 experiment shown in Figure S1. A representative picture is shown for day 1 before splitting and addition of 4 mM ethanol (panel A). Panels B, D, F and G show L1 larvae incubated in 4 mM ethanol at days 10, 14, 21 and 28, respectively. Panels C and E show worms incubated in M9 medium alone at days 10 and 14, respectively. L1 larvae incubated in M9 medium alone are dead at days 21 and 28. The scale bar in each panel corresponds to 20 µm. Microscope instrument settings were the same for all images except that exposure times ranged from 2 ms to 69 ms.
(TIF)

Figure S3 **Wild-type L1 larvae incubated in 1 mM deuterated ethanol have similar lifespan to larvae incubated in 1 mM non-deuterated ethanol.** Larvae were treated as described in Figure 1. Mantel-Cox logrank analyses showed that the p-value for the differences between the control and both ethanol samples was <0.0001; there is no significant difference between the survival in ethanol and deuterated ethanol (p = 0.72).
(TIFF)

Figure S4 **Fatty acid profile of wild-type L1 larvae incubated in ethanol or deuterated ethanol (Replicate 1 trial 1).** Starved L1 larvae were incubated in M9 only, M9+1 mM ethanol or M9+1 mM deuterated ethanol (C_2_D_5_OD) medium, under conditions described in Figure 1. For each condition, L1 larvae (70,000) were collected at three different time points: 1 day after hatch (before adding ethanol or deuterated ethanol) and 1 and 7 days after incubation. For each time point L1 larvae (33,000) were collected and fatty acid methyl esters were generated and analyzed as described in Figure 7. Panel A shows the chromatogram for L1 larvae 1 day after hatch and before ethanol was added. Panels B and E show the lipid profile for L1 larvae incubated in M9 for 1 and 7 days, respectively. Panels C and F show the lipid profile for L1 larvae incubated in M9+ethanol for 1 and 7 days, respectively. Panels D and G show the lipid profile for L1 larvae incubated in M9+deuterated ethanol for 1 and 7 days, respectively. Red arrows indicate the pre-eluting peak containing deuterated fatty acids.
(TIFF)

Figure S5 **Fatty acid profile of wild-type L1 larvae incubated in ethanol or deuterated ethanol over time (Replicate 1 trial 2).** See Figure S4 for information about this figure.
(TIFF)

Figure S6 **Fatty acid profile of wild-type L1 larvae incubated in ethanol or deuterated ethanol over time (Replicate 1 trial 3).** See Figure S4 for information about this figure.
(TIFF)

Figure S7 **Amino acids in which no detectable incorporation of deuterium from ethanol was found in starved L1 larvae.** The isotope distribution is shown for amino acids from the experiment described in Figure 10 that did not demonstrate incorporation of deuterium from deuterated ethanol.
(TIFF)

Table S1 **Summary of lifespan results: small concentrations of ethanol extend lifespan.**
(PDF)

Table S2 **Summary of statistics of lifespan results.** Lifespans for 75%, 50%, and 25% median survival are given with the number of independent experiments and the standard deviation (SD). P-values for the comparison to no ethanol controls are given from Student's t-test for two-tailed, unpaired samples.
(PDF)
