# Supplementary figures and images for: Caenorhabditis elegans Battling Starvation Stress: Low Levels of Ethanol Prolong Lifespan in L1 Larvae
Source: PLoS One. 2012 Jan 18;7(1):e29984. doi: 10.1371/journal.pone.0029984 (PMC3261173; doi:10.1371/journal.pone.0029984)

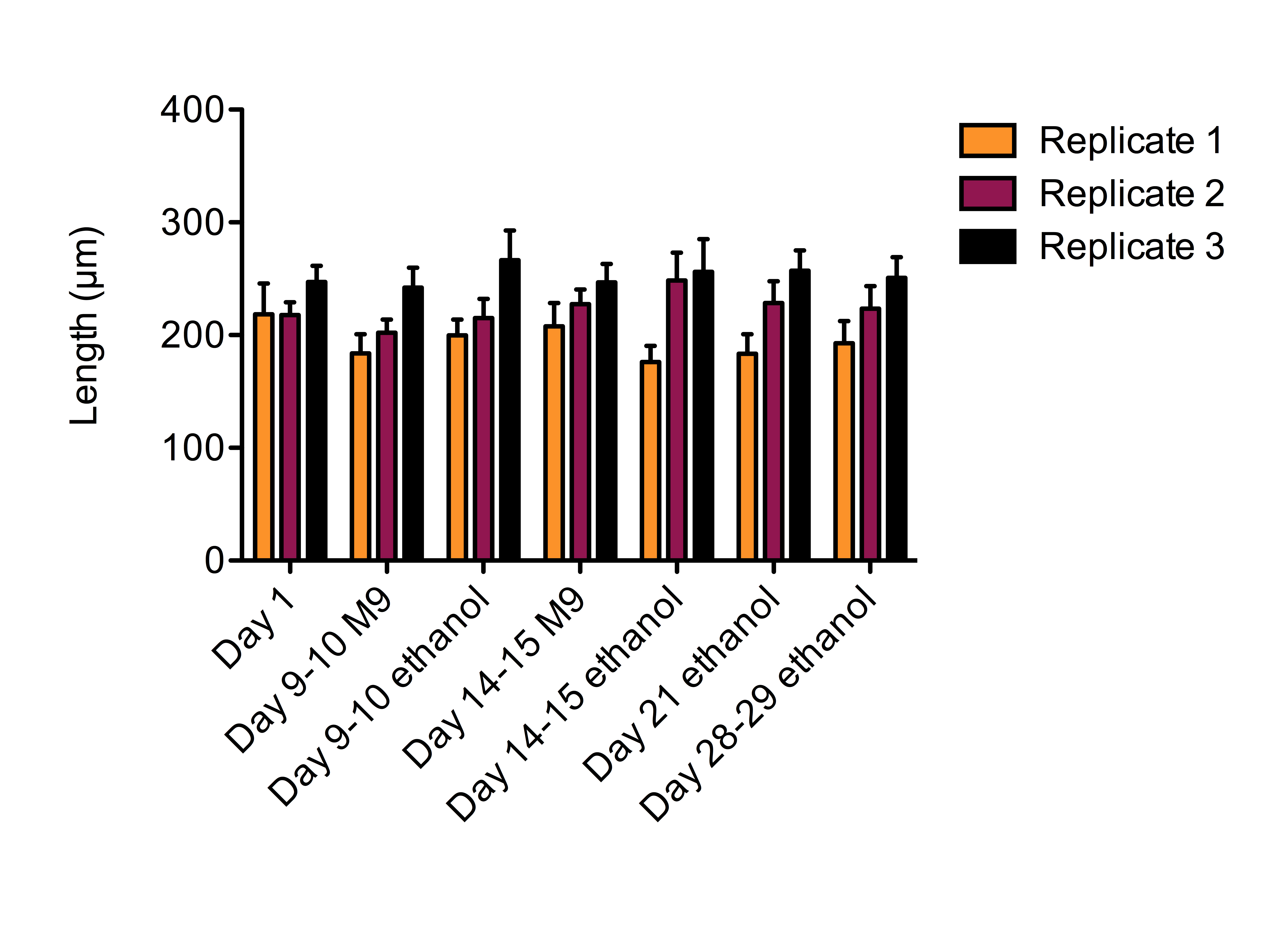

Supplement: Figure S1 — Starved L1 larvae incubated in the presence of ethanol do not progress to the L2 stage. To confirm the staging of L1 larvae, body length was measured by differential interference contrast microscopy using the ZEISS Axio Imager.M1 with Hamamatsu ORCA-ER digital camera C4742-80. For each condition and time point approximately 20 L1 larvae were measured using Volocity 5 5.3.2 software. In three separate replicates, L1 larvae starved in M9 medium were examined one day after hatching and then after incubation in M9 medium or M9 medium supplemented with 4 mM ethanol for 9–10 days, 14–15 days, 21 days, and 28–29 days. For day 21 and 28 or 29 only larvae incubated in ethanol were analyzed since larvae incubated in M9 were no longer alive. Previous work has shown that L1 larvae measure close to 250 µm [6], [21], [22]. (TIFF) [file pone.0029984.s001.tiff]

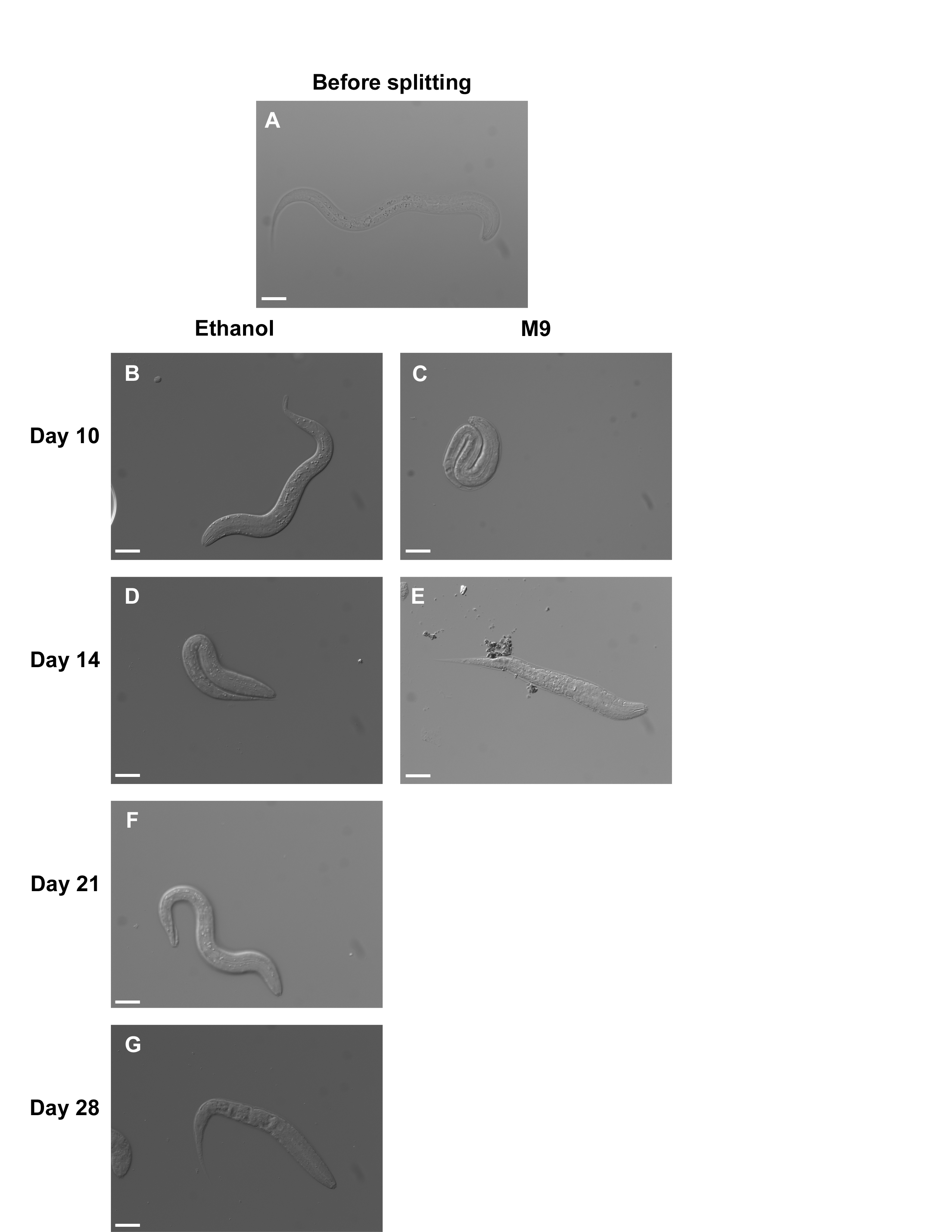

Supplement: Figure S2 — Wild-type L1 larvae incubated in 4 mM ethanol in M9 medium conserve healthy morphology longer than larvae starved in M9 medium alone. Micrographs are shown of the larvae from the replicate 1 experiment shown in Figure S1. A representative picture is shown for day 1 before splitting and addition of 4 mM ethanol (panel A). Panels B, D, F and G show L1 larvae incubated in 4 mM ethanol at days 10, 14, 21 and 28, respectively. Panels C and E show worms incubated in M9 medium alone at days 10 and 14, respectively. L1 larvae incubated in M9 medium alone are dead at days 21 and 28. The scale bar in each panel corresponds to 20 µm. Microscope instrument settings were the same for all images except that exposure times ranged from 2 ms to 69 ms. (TIF) [file pone.0029984.s002.tif]

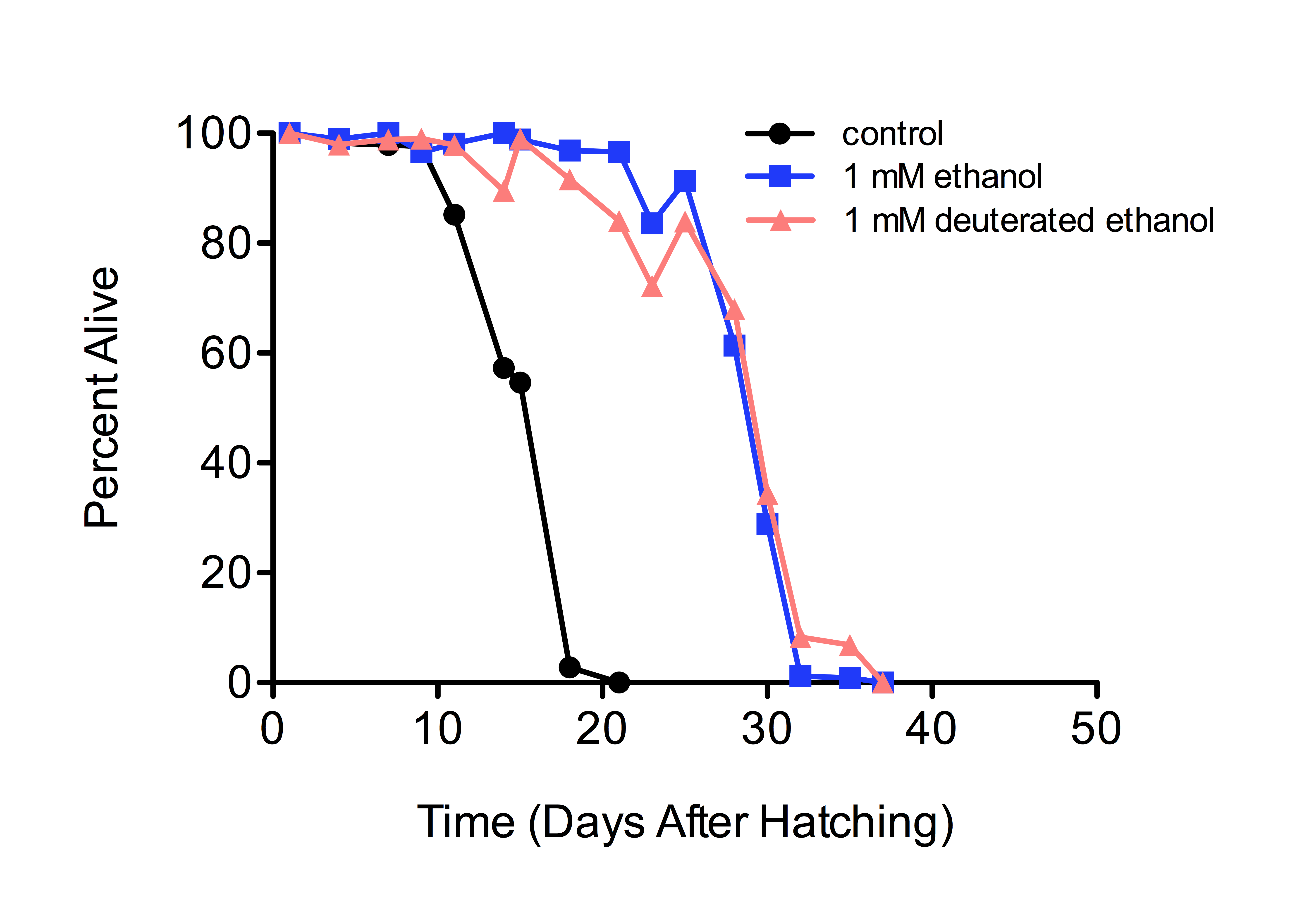

Supplement: Figure S3 — Wild-type L1 larvae incubated in 1 mM deuterated ethanol have similar lifespan to larvae incubated in 1 mM non-deuterated ethanol. Larvae were treated as described in Figure 1. Mantel-Cox logrank analyses showed that the p-value for the differences between the control and both ethanol samples was <0.0001; there is no significant difference between the survival in ethanol and deuterated ethanol (p = 0.72). (TIFF) [file pone.0029984.s003.tiff]

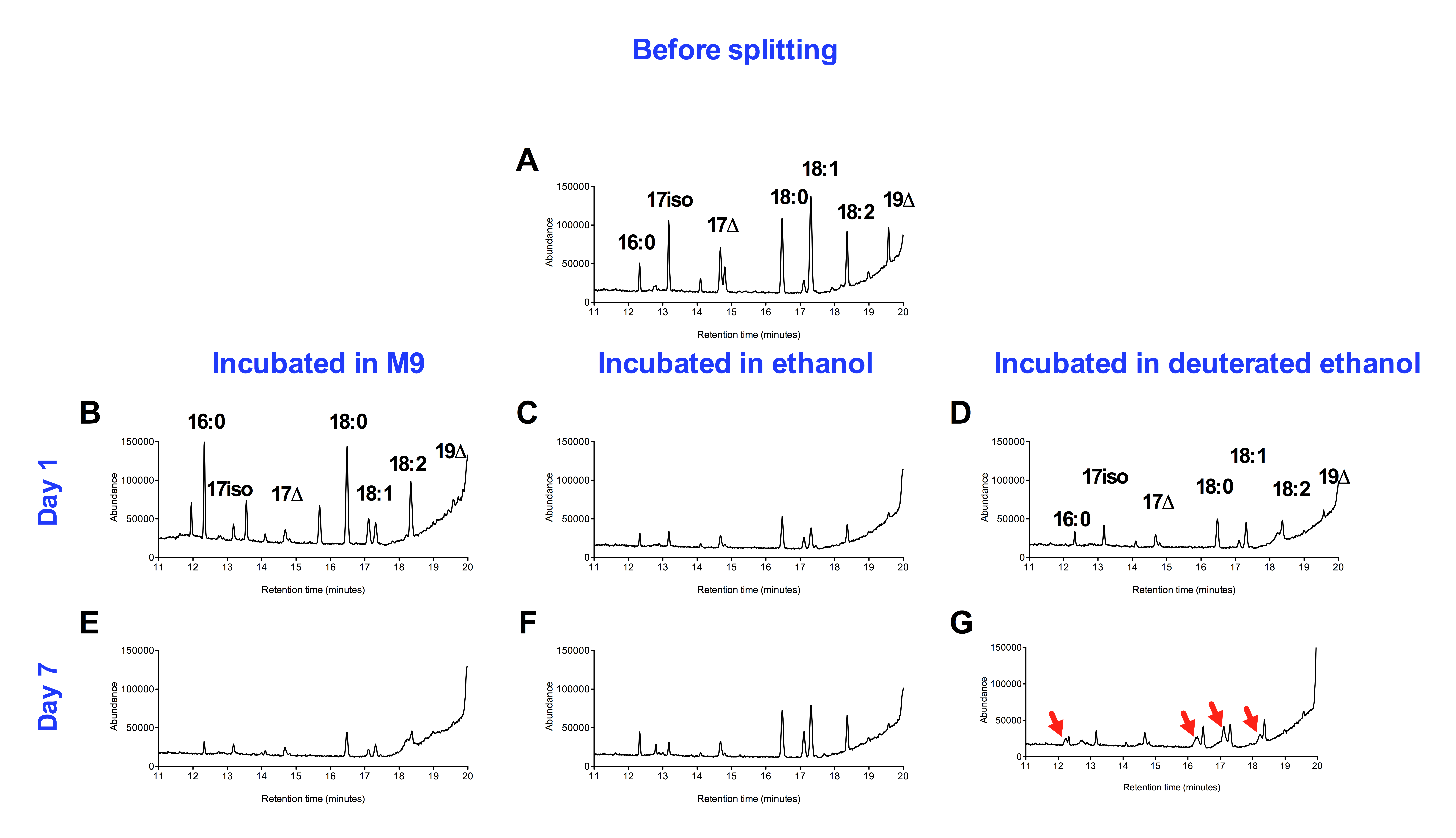

Supplement: Figure S4 — Fatty acid profile of wild-type L1 larvae incubated in ethanol or deuterated ethanol (Replicate 1 trial 1). Starved L1 larvae were incubated in M9 only, M9+1 mM ethanol or M9+1 mM deuterated ethanol (C2D5OD) medium, under conditions described in Figure 1. For each condition, L1 larvae (70,000) were collected at three different time points: 1 day after hatch (before adding ethanol or deuterated ethanol) and 1 and 7 days after incubation. For each time point L1 larvae (33,000) were collected and fatty acid methyl esters were generated and analyzed as described in Figure 7. Panel A shows the chromatogram for L1 larvae 1 day after hatch and before ethanol was added. Panels B and E show the lipid profile for L1 larvae incubated in M9 for 1 and 7 days, respectively. Panels C and F show the lipid profile for L1 larvae incubated in M9+ethanol for 1 and 7 days, respectively. Panels D and G show the lipid profile for L1 larvae incubated in M9+deuterated ethanol for 1 and 7 days, respectively. Red arrows indicate the pre-eluting peak containing deuterated fatty acids. (TIFF) [file pone.0029984.s004.tiff]

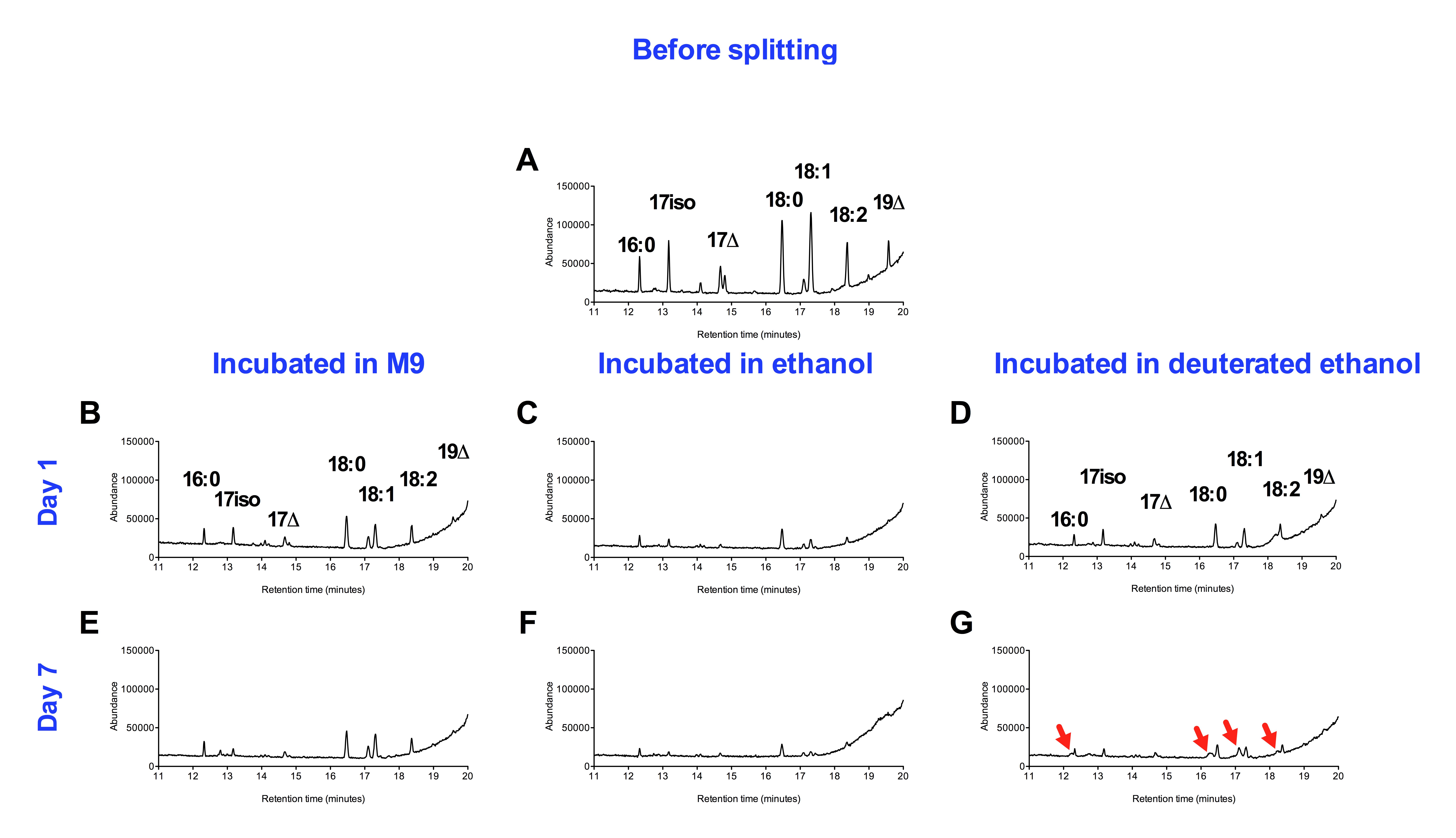

Supplement: Figure S5 — Fatty acid profile of wild-type L1 larvae incubated in ethanol or deuterated ethanol over time (Replicate 1 trial 2). See Figure S4 for information about this figure. (TIFF) [file pone.0029984.s005.tiff]

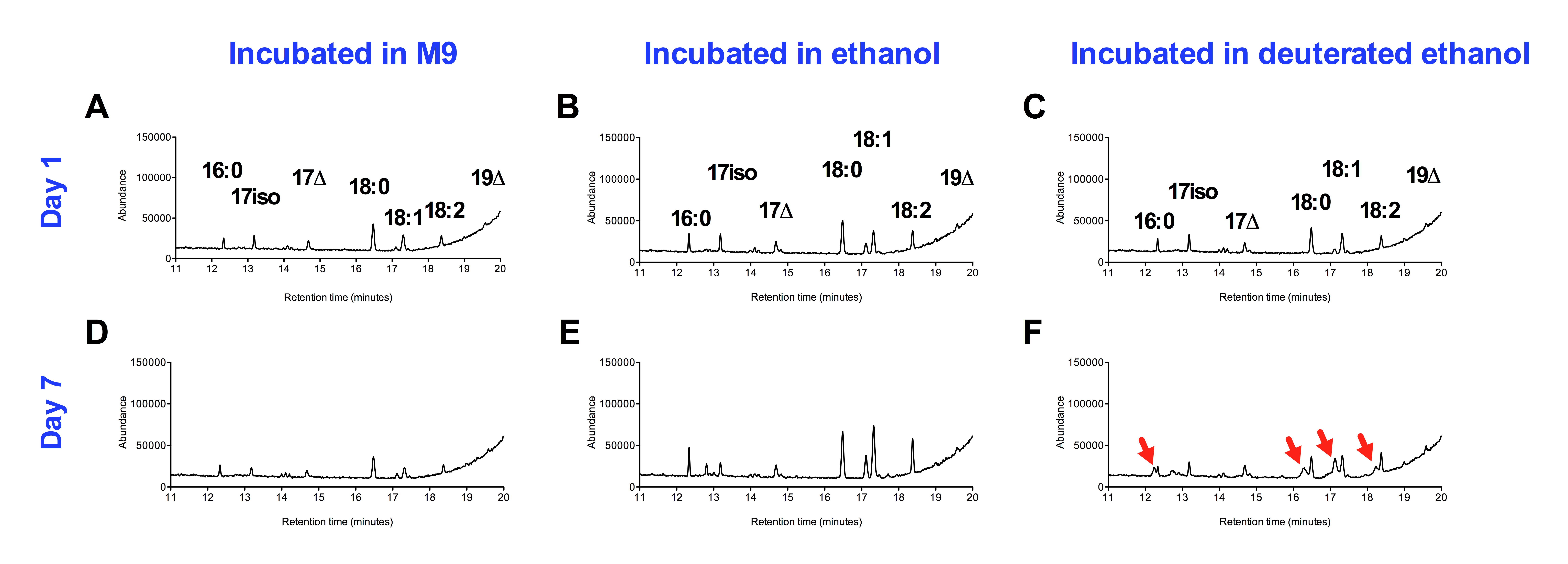

Supplement: Figure S6 — Fatty acid profile of wild-type L1 larvae incubated in ethanol or deuterated ethanol over time (Replicate 1 trial 3). See Figure S4 for information about this figure. (TIFF) [file pone.0029984.s006.tiff]
