# Supplementary material for: Caenorhabditis elegans Battling Starvation Stress: Low Levels of Ethanol Prolong Lifespan in L1 Larvae
Source: PLoS One. 2012 Jan 18;7(1):e29984. doi: 10.1371/journal.pone.0029984 (PMC3261173; doi:10.1371/journal.pone.0029984)
Supplement: Table S1 — Summary of lifespan results: small concentrations of ethanol extend lifespan. (PDF) [file pone.0029984.s008.pdf]

Data from Figure	strain	metabolite	75% survival (days)	50% survival (days)	25% survival (days)
1A	*pcm-1(qa201)*	none	9	10	12
1A	N2	none	9	10	12
1B	*pcm-1(qa201)*	17 mM ethanol	31	33	35
1B	N2	17 mM ethanol	29	34	38
1C	*pcm-1(qa201)*	17 mM ethanol + cholesterol	20	21	23
1C	N2	17 mM ethanol + cholesterol	29	32	35
2A	N2	none	10	12	13
2A	N2	4 mM ethanol	18	22	25
2A	N2	17 mM ethanol	19	22	25
2A	N2	68 mM ethanol	24	26	29
2B	N2	none	8	10	11
2B	N2	4 mM ethanol	22	26	29
2B	N2	17 mM ethanol	22	25	29
2B	N2	34 mM ethanol	23	26	27
2B	N2	68 mM ethanol	25	30	33
2C	N2	none	12	15	17
2C	N2	1 mM ethanol	27	29	30
2C	N2	0.1 mM ethanol	16	18	20
2C	N2	0.01 mM ethanol	11	15	17
2C	N2	0.001 mM ethanol	12	11	17
4	N2	none	13	15	17
4	N2	4 mM ethanol added day 1	21	27	34
4	N2	4 mM ethanol added day 5	24	28	31
4	N2	4 mM ethanol added day 7	23	25	33
4	N2	4 mM ethanol added day 10	18	25	32
4	N2	4 mM ethanol added day 13	13	15	21
5	*eat-2 (ad1116)*	none	9	10	11
5	*eat-2 (ad1116)*	4 mM ethanol	14	15	17
6A	N2	none	10	11	12
6A	N2	4 mM methanol	10	11	12
6A	N2	4 mM propanol	20	22	26
6A	N2	4 mM butanol	19	22	23
6B	N2	none	9	11	12
6B	N2	4 mM ethanol	22	25	27
6B	N2	4 mM methanol	7	10	12
6B	N2	4 mM propanol	20	21	22
6B	N2	4 mM isopropanol	10	11	12
6B	N2	4 mM butanol	21	24	30
6B	N2	4 mM isobutanol	20	21	25
