# Supplementary material for: Caenorhabditis elegans Battling Starvation Stress: Low Levels of Ethanol Prolong Lifespan in L1 Larvae
Source: PLoS One. 2012 Jan 18;7(1):e29984. doi: 10.1371/journal.pone.0029984 (PMC3261173; doi:10.1371/journal.pone.0029984)
Supplement: Table S2 — Summary of statistics of lifespan results. Lifespans for 75%, 50%, and 25% median survival are given with the number of independent experiments and the standard deviation (SD). P-values for the comparison to no ethanol controls are given from Student's t-test for two-tailed, unpaired samples. (PDF) [file pone.0029984.s009.pdf]

			75% survival (days)	50% survival (days)	25% survival (days)
**control (M9 medium, n=7)**		mean ± SD	10.2 ± 1.83	12.0 ± 2.28	13.3 ± 2.35
**control versus 4 mM ethanol (n = 4)**	**control**	mean ± SD	10.0 ± 2.2	11.8 ± 2.4	13.1 ± 2.5
	**4 mM ethanol**	mean ± SD	20.9 ± 2.0	25.0 ± 2.1	28.7 ± 3.7
		p-value	**0.00038**	**0.00017**	**0.00076**
**control versus 17 mM ethanol (n = 3)**	**control**	mean ± SD	9.1 ± 0.8	10.5 ± 1.0	11.8 ± 0.9
	**17 mM ethanol**	mean ± SD	23.3 ± 5.0	26.8 ± 6.1	30.7 ± 7.1
		p-value	**0.037**	**0.041**	**0.042**
**control versus 4 mM alcohols (n = 2)**	**control**	mean ± SD	9.4 ± 0.9	11.1 ± 0.2	12.3 ± 0.1
	**4 mM methanol**	mean ± SD	8.5 ± 2.6	10.9 ± 0.6	12.3 ± 0.1
		p-value	**0.71**	**0.79**	**0.63**
	**4 mM n-propanol**	mean ± SD	20.3 ± 0.2	21.7 ± 0.9	23.8 ± 2.6
		p-value	**0.030**	**0.033**	**0.10**
	**4 mM n-butanol**	mean ± SD	19.6 ± 1.4	23.1 ± 1.0	26.6 ± 4.7
		p-value	**0.020**	**0.031**	**0.15**
